# Differential Requirements for MCM Proteins in DNA Replication in *Drosophila* S2 Cells

**DOI:** 10.1371/journal.pone.0000833

**Published:** 2007-09-05

**Authors:** Gilles Crevel, Reina Hashimoto, Sharron Vass, Jake Sherkow, Masamitsu Yamaguchi, Margarete M.S. Heck, Sue Cotterill

**Affiliations:** 1 Basic Medical Sciences, St. George's University of London, London, United Kingdom; 2 Department of Applied Biology, Insect Biomedical Research Center, Kyoto Institute of Technology, Kyoto, Japan; 3 Queen's Medical Research Institute Centre for Cardiovascular Science, College of Medicine and Veterinary Medicine, University of Edinburgh, Edinburgh, United Kingdom; University of Minnesota, United States of America

## Abstract

**Background:**

The MCM2-7 proteins are crucial components of the pre replication complex (preRC) in eukaryotes. Since they are significantly more abundant than other preRC components, we were interested in determining whether the entire cellular content was necessary for DNA replication in vivo.

**Methodology/Principle Findings:**

We performed a systematic depletion of the MCM proteins in *Drosophila* S2 cells using dsRNA-interference. Reducing MCM2-6 levels by >95–99% had no significant effect on cell cycle distribution or viability. Depletion of MCM7 however caused an S-phase arrest. MCM2-7 depletion produced no change in the number of replication forks as measured by PCNA loading. We also depleted MCM8. This caused a 30% reduction in fork number, but no significant effect on cell cycle distribution or viability. No additive effects were observed by co-depleting MCM8 and MCM5.

**Conclusions/Significance:**

These studies suggest that, in agreement with what has previously been observed for Xenopus in vitro, not all of the cellular content of MCM2-6 proteins is needed for normal cell cycling. They also reveal an unexpected unique role for MCM7. Finally they suggest that MCM8 has a role in DNA replication in S2 cells.

## Introduction

The MCM (minichromosome maintenance) 2–7 proteins play an important role in DNA replication in eukaryotes. They are involved during initiation where they are needed to form the preRC (pre-Replicative Complex) (reviewed [1]). This complex is formed at origins of replication by the sequential binding of ORC1-6, cdc6, cdt1 and MCM2-7, and is absolutely required for all subsequent processes of replication. The MCM proteins are also proposed to act during elongation as the replicative helicase (reviewed [2]).

In archael species which have a single MCM protein, the active complex has been suggested to be a hexamer or double hexamer (reviewed [3]. The MCM2-7 proteins can also form hexamers [4] [5] [6]. Several studies have mapped subunit arrangement in the hexamer [7] [8] [9] , however the catalytic constituents of the active complex are not yet determined. Although all six budding yeast MCMs are required for replication [10], it is not clear if they all participate directly in the catalysis of the helicase reaction. In mouse and *S. pombe* active helicases have been isolated containing only MCM4/6/7 [11] [12]. Studies in Drosophila have also suggested that cdc45 and the GINS complex may be necessary for helicase activity [13]. Temporal differences in chromatin loading of individual MCM proteins [6] [14] also suggest differential function of MCM proteins. Recently a new MCM family member (MCM8) has been isolated [15] and shown to form a homohexameric helicase. Functional studies have suggested a role for MCM8 in elongation in Xenopus [16] or preRC formation in human cells [17].

Most preRC proteins are present at low levels, however the MCMs are relatively abundant (>40 complexes per origin in Xenopus [18] [19]. Their involvement in both initiation and elongation might necessitate larger amounts of protein. Consistent with this, depletion of MCM proteins using degron constructs in *S. cerevisiae* produces an S phase block [10]. Similar effects are reported on reduction of MCM4 in human cells [14]. Such observations have prompted models where multiple MCM complexes co-operate to give helicase activity [20] [19]. By contrast, in *Xenopus* extracts a 90% reduction of MCM binding at origins still permits efficient in vitro replication [21]. Depletion of MCM7 in human cells [22] [23] and MCM 3 and 5 in Drosophila Kc cells [24] are also reported to have no effect on replication. The extra MCM proteins do however seem to be needed for the recovery of replication in Xenopus extracts in vitro in the presence of inhibitors of ATR [25]. In addition others have suggested that some of the MCM content may be involved in processes other than replication (reviewed [26] ).

Detailed analysis of the MCM paradox has so far only been carried out in detail in vitro in cell free extracts. We were interested to see if the same effects were observed in vivo. Here we report on the cellular effects of systematic depletion of MCM2-8 in *Drosophila* S2 cells. We present data supporting the hypothesis that some of the cellular MCM content may be redundant in the normal cell cycle. We also present evidence that lends further weight to the suggestion that not all MCM proteins have equivalent cellular roles. Finally our data also suggests that, as in other organisms, Drosophila MCM8 has a role in DNA replication.

## Results

### *Drosophila* MCM mutants

To determine whether we could observe differential cellular requirements for MCM proteins we compared the phenotypes of 4 MCM2/4/6 mutants (*MCM2^rl74^*, *dpa^1^* [MCM4], *MCM6^k1214^* and *MCM6^3^*) (Table 1). As reported earlier [27] [28] [29], all lines showed loss or severe reduction in size of imaginal discs. D*pa^1^* and *MCM6^3^* also showed a reduction in BrdU incorporation in larval brains. Since *dpa^1^* had been reported to exhibit defective mitosis we examined all of the *MCM* mutants for mitotic index and phenotype. Only *dpa^1^* showed a significantly higher mitotic index than control cells (57% higher). The mitotic index in *MCM6^3^* was comparable to control cells, while decreases were seen for *MCM6^k1214^* and *MCM2^rl74^* (20% and 26% lower) (table 1).

**Table 1:** Quantitation of cell cycle parameters of Drosophila MCM mutants.

Mutation	S phase Index	Mitotic Index	Mitotic Cells
Wild type	14.7%	1%	normal
*mcm2^rl74^*	*ND*	0.7% (⇓ by 26%)	normal
*dpa^1^* [mcm4]	10.3% (⇓ by 30%)	1.5% (⇑ by 57%)	abnormal
*mcm6^3^*	9.1% (⇓ by 38%)	1% (similar to wt)	normal
*mcm6^k1214^*	*ND*	0.8% (⇓ by 20%)	normal

**Figure 1:**
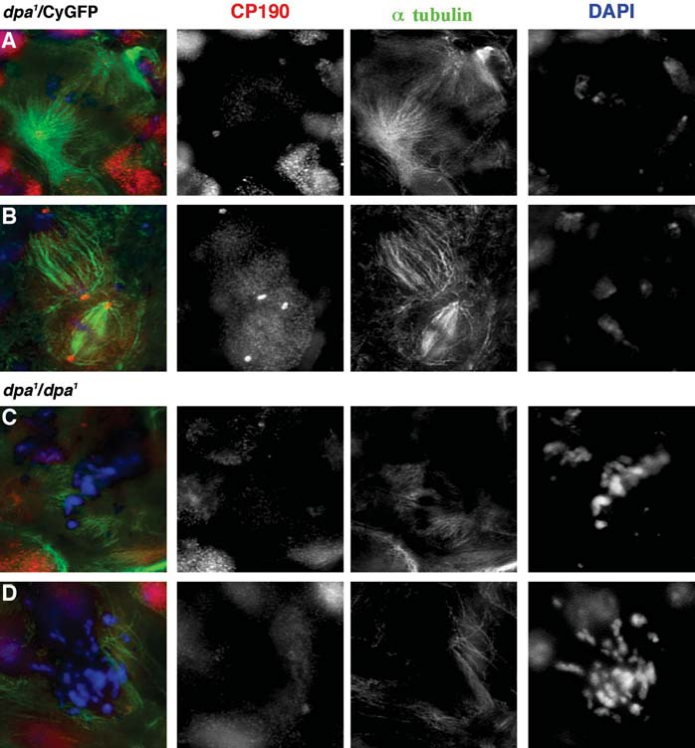
Dpa^1^ mutants show abnormal chromosome condensation and unorganised mitotic spindles. Neuroblasts of heterozygous or homozygous third instar larvae (prepared as in 4) were stained for: centrosomes (red) using CP190 antibodies (a kind gift from Will Whitfield) and Goat anti-rabbit TxRed (Jackson labs); spindles (green) using anti-tubulin (Clone DM1A-Sigma) and goat anti-mouse Alexa 488 (Jackson Labs); and DNA (blue) using DAPI (Sigma). Heterozygous neuroblasts showed normal spindles with correct localization of CP190 at the poles in metaphase (A/B) and anaphase (B), and normally condensed chromosomes. Homozygous neuroblasts (C/D) showed fragmented, hyper-condensed and aneuploid chromosomes, and disorganised mitotic spindles usually lacking CP190.

Not surprisingly, *MCM6^3^* cells appeared to progress normally through mitosis (not shown). Despite *MCM2^rl74^* and *MCM6^k1214^* having fewer mitotic cells, no mitotic aberrations were detected, suggesting that their defect was a pre-mitotic delay. For *dpa^1^* however the defect seemed to be due to a metaphase delay (anaphases were absent). While chromosome condensation was unaffected, mitotic chromosomes appeared broken and did not congress to a metaphase plate (Figure 1). Spindle defects were also seen. Microtubules appeared to radiate towards the chromosomes, but without apparent order. In addition roughly half of the *dpa^1^* mutant cells showed abnormal centrosome number or fragments (Figure 1).

### Depletion of MCM2-7 proteins by dsRNA-interference

**Figure 2:**
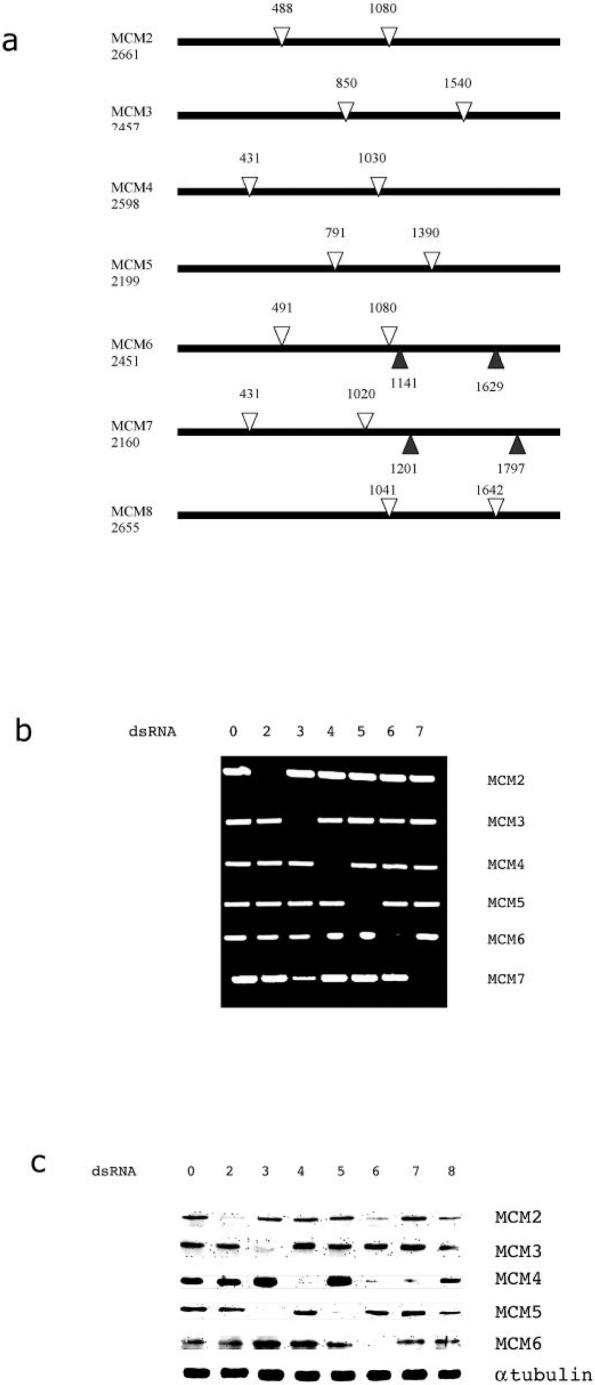
a. Schematic detailing the regions of each MCM targeted for dsRNA. Base positions are quoted relative to the start AUG. Shaded arrows designate the 2^nd^ dsRNA made from MCM6/7. b. Agarose gel of RT-PCR products showing the effects of RNAi depletion of individual MCMs on MCM mRNA expression levels. Horizontal numbers designate the MCM targeted by RNAi. Vertical numbers show the RT PCR target. The data presented is one complete representative data set from multiple repetitions. c. Western blot to show the effect of RNAi depletion of individual MCMs on MCM protein expression levels in total cell extracts. Horizontal numbers designate the MCM targeted by RNAi. Vertical numbers show the antibody used for western blotting. Tubulin expression is included as a loading control. The data presented is one complete representative data set from multiple repetitions.

The mutant analysis suggests differential requirements for individual MCM proteins, however interpretation was complicated by the fact that individual MCM mutant lesions were of different types (*MCM2^rl74^* is a P element insertion, *dpa^1^*, *MCM6^k1214^* and *MCM6^3^* are point mutations). We therefore decided that a more systematic approach would be to deplete each MCM protein using dsRNA-mediated interference (RNAi) in *Drosophila* S2 cells. A 500–600 bp dsRNA region was produced for each cDNA (Figure 2a) and introduced into S2 cells. RT-PCR was used to monitor loss of mRNA. For each MCM some loss of mRNA was observed as early as 24 h (not shown) and levels decreased further until after 96h no mRNA was detectable (Figure 2b). This depletion was specific since in each case only the mRNA for the targeted MCM was reduced (Figure 2b).

Loss of mRNA correlated with a decrease in protein level for the targeted protein (Figure 2c). For MCM2-6 all proteins were decreased to <5% of the normal level, and some were no longer detectable. MCM7 levels were also decreased but precise quantitation of the extent of the decrease was less accurate due to the relatively low sensitivity of the MCM7 antibody (not shown).

### Loss of MCM3, 6 and 7 causes instability of other MCM proteins

Figure 2c shows that for MCM2/4/5 only the targeted protein was depleted. However for MCM3/6/7 additional instabilities were consistently observed. In cells depleted of MCM3 MCM5 was undetectable. For MCM6 depleted cells reductions were seen in MCM2 (∼75%) and MCM4 (∼90%). Finally in MCM7 depleted cells MCM4 was reduced by ∼90%. It is unlikely that the changes were due to cross-reaction of the dsRNA reagents since no corresponding reduction was seen in mRNA levels (Figure 2b). In addition a different target region of MCM6 also caused MCM4 reduction (data not shown). The observed instability does not appear to take place via the proteosome complex since the addition of proteosome inhibitors does not prevent the protein co-depletion (data not shown).

**Figure 3:**
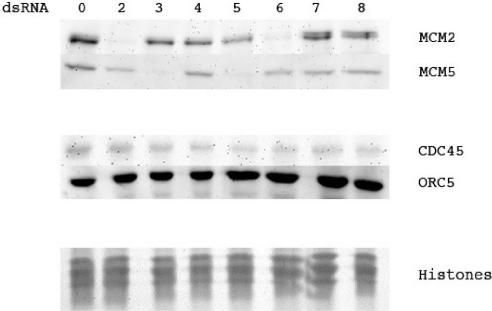
Immunoblot to show the effect of MCM depletions on the levels of MCM2, MCM5 ORC5 and CDC45 bound to chromatin. Coomassie stained histones are included as a loading control. The amount of each protein was compared to the histones using Alpha Innotech imager software. Horizontal numbers show the MCM protein targeted by RNAi. Several repetitions of this experiment were performed and this represents 1 complete data set.

In *S. cerevisiae,* depleting one MCM protein prevents other complex members from associating with chromatin [30]. To determine whether an analogous situation occurred in *Drosophila,* control and MCM-depleted cells were fractionated into chromatin bound and soluble fractions and the location of non-targeted MCMs analysed. In all cases only proteins depleted as a result of RNAi treatment showed reduced chromatin association. Results for MCM2 and MCM5 are shown in Figure 3. The binding of the MCMs is considerably reduced by the treatment of the chromatin with DNase suggesting that this is true chromatin binding rather than non specific binding (data not shown). Therefore in S2 cells the depletion of one MCM protein does not prevent binding of other members of the complex to chromatin. We further checked whether the reduction in MCM proteins had any effect on the binding of other replication proteins. Figure 3 also shows that neither dORC5 or dCDC45 binding were affected by depletion of any of the MCM proteins.

### MCM7 depletion causes an S phase defect

**Figure 4:**
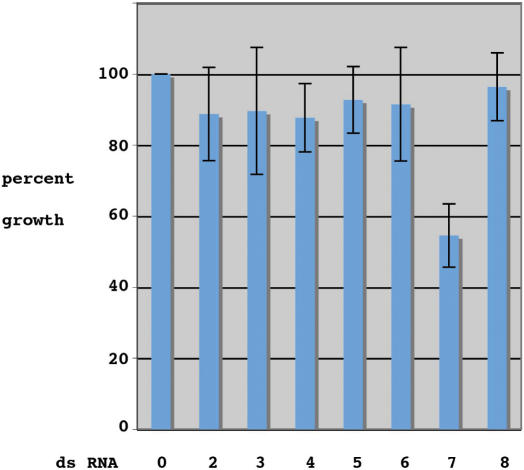
Day 5 growth profiles of MCM targeted cells compared to a control cell line. The data presented shows combined data from seven independent experiments. The data is expressed as a percentage of the control. Lane 1 is the control and lanes 2-8 are MCM 2-8 resepectively.

Since we had been able to significantly reduce MCM protein levels, we proceeded to analyse the cellular effects of the reductions. We initially examined the growth rate of MCM-depleted cells compared to mock-treated cells (no RNA or unrelated dsRNA produced the same results). The combined results of seven such experiments are shown in Figure 4. Surprisingly, for MCM2-6 reductions in protein level had no statistically significant effect on cell doubling rates. However MCM7 depleted cells consistently showed a significant decrease in cell number. Total cell numbers after 5 days for these cells were ∼50% of that for wild type cells (Figure 4), this fell to 30% after 7 days (data not shown). It is likely that the observed effect was specific to MCM7 since dsRNA for a different region of MCM7 gave the same result (not shown).

**Figure 5:**
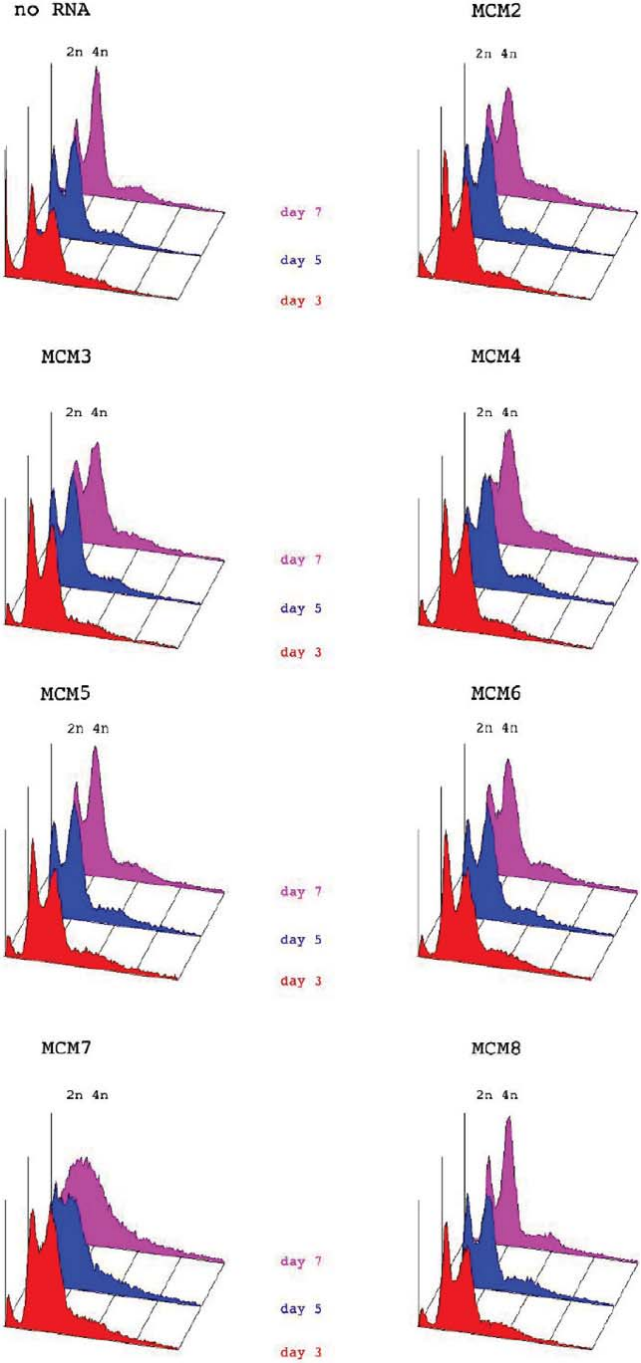
FACS analysis profiles of cells targeted with dsRNA against MCM2-7 proteins as compared to control targeted cells. The data presented is one complete representative data set from multiple repetitions.

We also analysed the cell cycle distribution of MCM2-7 depleted cells using FACS analysis. A representative data set is shown in Figure 5. Compared to mock depleted cells, no reproducible changes were seen in the DNA content profiles for cells with reduced levels of MCM2-6. In contrast, MCM7 depleted cells reproducibly showed a broad peak of DNA content between G1 and G2, suggesting that they had difficulty in traversing S phase.

### RNAi depletion of MCM8 in S2 cells

**Figure 6:**
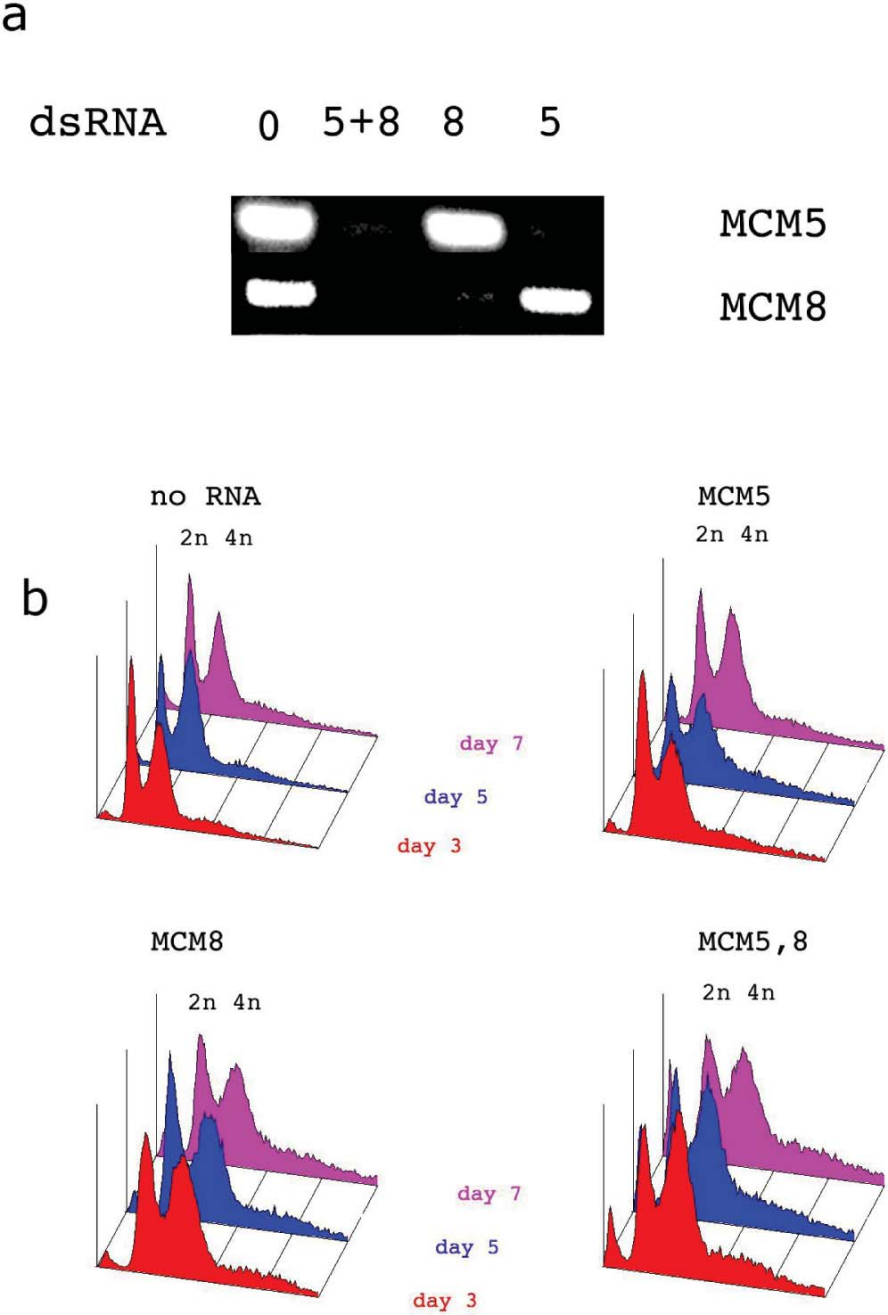
a. Agarose gel of RTPCR products to show the effects of RNAi depletion of MCM5 and MCM8 and MCM 5 plus 8 on the MCM5 and 8 levels. b. FACS profiles of cells targeted with dsRNA against MCM8, and MCM5 plus MCM8. The data presented is one complete representative data set from multiple repetitions.

The lack of effects seen after MCM2-6 depletion could have been due to compensation by a functionally redundant protein. Since a possible candidate for this was MCM8 we targeted *Drosophila* MCM8 [31] with dsRNA (Figure 2a). We were able to decrease MCM8 mRNA to undetectable levels by this procedure (Figure 6a). Depletion of MCM8 alone did not significantly affect cellular levels of MCM2-7 (Figure 2c), the binding of MCM2, MCM5, dORC5 or dCDC45 to chromatin (Figure 3), cell doubling rate (Figure 4) or cell cycle distribution (Figure 5).

To determine whether MCM8 could compensate for the loss of MCM2-6 we performed RNAi simultaneously targeting MCM5 and MCM8. Figure 6a shows that the mRNA for both proteins was reduced to undetectable levels. Even this treatment however had no significant effect on the growth rate (not shown) or cell cycle distribution of S2 cells (Figure 6b). MCM8 also showed no additional effect on the viability and cell cycle parameters observed for MCM7 depleted cells ( data not shown).

### Effect of MCM protein loss on PCNA loading

**Figure 7:**
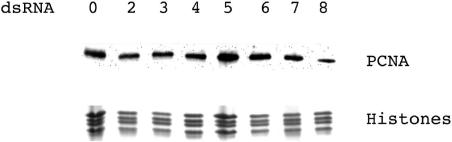
Immunoblot to show the effect of MCM depletions on the levels of PCNA bound to chromatin. Coomassie stained histones are included as a loading control. The amount of PCNA was compared to the histones using Alpha Innotech imager software. Horizontal numbers show the MCM protein targeted by RNAi.

Lower MCM protein levels could result in less MCM at each replication fork or a reduced number of active forks. The amount of PCNA associated with chromatin has previously been used as a measure of the number of active forks in a cell [14]. We therefore used fractionation and immunoblotting to compare the level of PCNA bound to chromatin in mock- and MCM-depleted cells. None of the treatments altered the total cellular PCNA content (not shown). Depletion of MCM2-7 did not change the amount of chromatin-associated PCNA (Figure 7). This suggests that a >95% loss of MCM2-7 does not produce a comparable reduction in active replication forks.

Depletion of MCM8 consistently reduced PCNA binding by 30–50%. This suggests a role for MCM8 protein in DNA replication in *Drosophila* cells. It further suggests that S2 cells can tolerate a reduction in the number of active replication forks without a significant effect on the cell cycle distribution or cell viability.

## Discussion

We have used RNAi in *Drosophila* S2 cells to efficiently deplete the MCM2-8 proteins. The data produced supports three main conclusions about MCM proteins in this system.

Firstly, although we could demonstrate specificity of the RNAi depletions, we observed co-instability for certain combinations of MCM proteins. Some of our observations can be explained based on the composition of reported MCM sub-complexes (reviewed [2]). Therefore, a reduction in MCM5 when MCM3 is targeted, and MCM4 when MCM6 and MCM7 are targeted might be related to loss of stability of the MCM3/5 and MCM4/6/7 complexes. However this is not a complete explanation since MCM3 stability is unaffected by MCM5 depletion and MCM6/7 are not affected by depletion of other MCM4/6/7 components. In addition, MCM2, which is affected by MCM6 depletion, is not thought to be a component of either complex. We can also not rationalise the co-reductions based on models proposed by us or others for the structure of the MCM hexameric complex [7] [8] [9]. Although we do not understand the basis for the co-reductions we were able to show that they did not occur via the proteosome pathway since treatment with proteosome inhibitors had no effect. Co-instability of MCM proteins has been reported in other systems, but the reported combinations differ from those we observe in *Drosophila* [23].

Secondly our data suggest that a dramatic reduction in the level of MCM2-6 and 8 in vivo in *Drosophila* S2 cells has little apparent effect on cell survival and DNA replication. This therefore suggests that the MCM paradox–originally observed in Xenopus cell free extracts [21] can also be observed in vivo for Drosophila. Cell viability has also previously been shown to be unaffected for MCM2 and 5 depletions in Drosophila Kc cells [24]. Whether the same effects will also be seen in other higher eukaryotes is unclear and in fact it has been reported that human cells cannot traverse S if MCM4 is depleted [14]. Since it is unlikely that the lack of replication effects on depletion of MCM2-6 is due to a different role for the MCM complex in *Drosophila* we suggest two other possibilities. Firstly, consistent with what has been suggested for Xenopus it might be that under normal circumstances most of the MCM protein in cells is redundant. We estimate that there are 50–100 MCM complexes per origin (assuming origin spacing of 40–100 kb) in S2 cells. Therefore even cells which have lost 99% of a specific MCM should have enough protein to ensure that most origins have one MCM complex. A single MCM complex per origin may therefore be sufficient to allow a full complement of activated replication forks as measured by PCNA loading. In this case our results support proposed MCM mechanisms involving single or double hexamers [32] rather than those that require bulk chromatin loading of MCMs [19] [20]. Perhaps in support of this suggestion we do not see effects of the MCM depletion on cdc45 chromatin loading. Cdc45 has been suggested to form an active component of the replicative helicase complex with GINS and MCM proteins [13]. It is therefore possible that despite the drastic reduction in the total number of MCM complexes in the dsRNA treated S2 cells the total number of active helicases has not altered.

The second possibility is that MCM loss is compensated for by other proteins. We investigated whether MCM8 could perform this function. The decrease in PCNA loading observed on depletion of MCM8 suggests that *Drosophila* MCM8 does play a role in replication. From our data the exact nature of its role is unclear, however the lack of an effect of the depletion on cdc45 loading suggests that unlike the MCM2-7 proteins it is unlikely to be required for the loading of downstream initiation factors. In addition MCM8 cannot be the MCM2-6 compensating protein since co-depletion of MCM5 and MCM8 does not synergistically affect cell viability or DNA replication.

Finally the differences observed between depletion of MCM7 and MCM2-6 suggest that not all MCM proteins are equivalent. The mechanism behind this differential behaviour is not clear. Although a complex of MCM4/6/7 has been shown to have helicase activity, there is no evidence that MCM7 acts independently. Therefore MCM7 may have additional cellular functions. A role for MCM7 as a damage sensor via the ATR pathway has been suggested by work in human [23] and *S. cerevisiae* [22] cells where depletion or mutation of MCM7 produces cells defective in the UV-induced S-phase checkpoint. In *Xenopus* extracts MCM7 has also been shown to bind to the Rb protein [33] leading to a brake on DNA replication. It is possible that the MCM7 effect that we observe is related to a failure of a negative control. This could lead to more significant damage which activates other checkpoints to cause the S phase stop. How this might be related to the roles of the S.cerevisiae and human MCM7 protein in the UV checkpoint is unclear since RNAi depletion of human MCM7 was not reported to show this effect [14]. The level of MCM7 protein remaining after depletion is significantly higher in human than *Drosophila* cells, however less efficient depletion of Drosophila MCM7 has been seen to produce the same effect (data not shown). Alternatively in addition to acting as a negative regulator of replication, MCM7 may have a positive regulatory effect on replication. In either case the effect is likely to involve MCM7 directly, rather than occurring as a secondary effect of a replication defect, since similar effects are not observed for other MCM proteins.

## Materials and Methods

### Fly stocks

all fly stocks (canton s (wild type), *mcm2^rl74^*, *dpa^1^*, *mcm6^3^*, and *mcm6^k1214^* were obtained from the bloomington stock center.

### Antibody reagents

MCM antibodies were described previously [7]. Antibodies against PCNA and CP-190 were kind gifts from Paul Fisher and Will Whitfield. DAPI and anti-tubulin antibodies (Clone DM1A) were from Sigma. Goat anti-rabbit TxRed, and goat anti-mouse Alexa 488 were from Jackson Labs.

### Immunofluorescence staining of larval brains

preparation, visualisation and quantification of mitotic and s-phase indices were as described previously [34]

### Western blotting

Proteins from SDS PAGE were blotted onto Hybond ECL (Amersham) and developed with Supersignal West Pico (Pierce). Visualisation and quantitation were carried out using an Alpha Innotech gel documentation system.

### dsRNA-mediated interference

Sequence specific primers each containing a 5′ T7 RNA polymerase binding site were designed for MCM2-8 (Fig.ô 2A). The dsRNA was made using MEGAscript T7 kit (Ambion) as per manufacturers instructions. The RNAi experiment was carried out on S2 cells in exponential growth phase as described [35]. 15 µg of RNA was added per well (10^6^ cells) and the cells were monitored by cell count, FACS analysis, western blotting and RT-PCR over a period of 7 days.

### RT-pcr

cDNA from 500,000 S2 cells was made using the cell to cDNA kit (Ambion) as per manufacturers instructions with the following modifications: cells were lysed in 100 µl lysis buffer and the RNAase inactivated by heat treatment. 20 µl of the lysate were used for the reverse transcription reaction. The equivalent of 5,000 cells (1 µl of the reverse transcription reaction) was amplified using Megamix blue (Microzone). Amplified fragments were run on agarose gels and visualised with EtBr.

Amplified regions were located at the following nucleotide positions relative to the start ATG: MCM2 1381–1550; MCM3 1701–1869; MCM4 1261–1400; MCM5 481–630; MCM6 781–922; MCM7 321-480 and MCM8 311–450.

### Flow cytometry

cells were harvested and fixed using ethanol. Immediately prior to use they were treated with 10ug/ml RNase/1mM EDTA and the DNA was stained with propidium iodide. Flow cytometry was carried out on an EPICS Xl (Coulter Beckman) using EXPO 32 adc software.

### Cell fractionation

Cell fractionation was carried out as described [35]. Samples were analysed by PAGE and western blotting. For the DNAse treatment of the chromatin, S2 cells were pelleted, washed in PBS and homogenised in a potter homogeniser using a tight pestle. The pellet was washed in PBS containing 0.1% triton, 5mM MgCl2 and protease inhibitors (complete EDTA free, Roche). They were resuspended in the same buffer at a concentration of 5×10^5^ cells/ml. DNAse (Turbo DNAse, Ambion) was added at a concentration of 100 units/ml. The incubation was for 1 hour on ice.

## References

[1] Kearsey SE, Cotterill S (2003). Enigmatic variations: divergent modes of regulating eukaryotic DNA replication.. Mol Cell.

[2] Labib K, Diffley JF (2001). Is the MCM2-7 complex the eukaryotic DNA replication fork helicase?. Curr Opin Genet Dev.

[3] Kelman Z, Hurwitz J (2003). Structural lessons in DNA replication from the third domain of life.. Nat Struct Biol.

[4] Lee JK, Hurwitz J (2000). Isolation and characterization of various complexes of the minichromosome maintenance proteins of Schizosaccharomyces pombe.. J Biol Chem.

[5] Prokhorova TA, Blow JJ (2000). Sequential MCM/P1 subcomplex assembly is required to form a heterohexamer with replication licensing activity.. J Biol Chem.

[6] Maiorano D, Lemaitre JM, Mechali M (2000). Stepwise regulated chromatin assembly of MCM2-7 proteins.. J Biol Chem.

[7] Crevel G, Ivetic A, Ohno K, Yamaguchi M, Cotterill S (2001). Nearest neighbour analysis of MCM protein complexes in Drosophila melanogaster.. Nucleic Acids Res.

[8] Davey MJ, Indiani C, O'Donnell M (2003). Reconstitution of the Mcm2-7p heterohexamer, subunit arrangement, and ATP site architecture.. J Biol Chem.

[9] Schwacha A, Bell SP (2001). Interactions between two catalytically distinct MCM subgroups are essential for coordinated ATP hydrolysis and DNA replication.. Mol Cell.

[10] Labib K, Tercero JA, Diffley JF (2000). Uninterrupted MCM2-7 function required for DNA replication fork progression.. Science.

[11] Lee JK, Hurwitz J (2001). Processive DNA helicase activity of the minichromosome maintenance proteins 4, 6, and 7 complex requires forked DNA structures.. Proc Natl Acad Sci U S A.

[12] You Z, Komamura Y, Ishimi Y (1999). Biochemical analysis of the intrinsic Mcm4-Mcm6-mcm7 DNA helicase activity.. Mol Cell Biol.

[13] Moyer SE, Lewis PW, Botchan MR (2006). Isolation of the Cdc45/Mcm2-7/GINS (CMG) complex, a candidate for the eukaryotic DNA replication fork helicase.. Proc Natl Acad Sci U S A.

[14] Ekholm-Reed S, Mendez J, Tedesco D, Zetterberg A, Stillman B (2004). Deregulation of cyclin E in human cells interferes with prereplication complex assembly.. J Cell Biol.

[15] Gozuacik D, Chami M, Lagorce D, Faivre J, Murakami Y (2003). Identification and functional characterization of a new member of the human Mcm protein family: hMcm8.. Nucleic Acids Res.

[16] Maiorano D, Cuvier O, Danis E, Mechali M (2005). MCM8 is an MCM2-7-related protein that functions as a DNA helicase during replication elongation and not initiation.. Cell.

[17] Volkening M, Hoffmann I (2005). Involvement of human MCM8 in prereplication complex assembly by recruiting hcdc6 to chromatin.. Mol Cell Biol.

[18] Mahbubani HM, Chong JP, Chevalier S, Thommes P, Blow JJ (1997). Cell cycle regulation of the replication licensing system: involvement of a Cdk-dependent inhibitor.. J Cell Biol.

[19] Edwards MC, Tutter AV, Cvetic C, Gilbert CH, Prokhorova TA (2002). MCM2-7 complexes bind chromatin in a distributed pattern surrounding the origin recognition complex in Xenopus egg extracts.. J Biol Chem.

[20] Laskey RA, Madine MA (2003). A rotary pumping model for helicase function of MCM proteins at a distance from replication forks.. EMBO Rep.

[21] Oehlmann M, Score AJ, Blow JJ (2004). The role of Cdc6 in ensuring complete genome licensing and S phase checkpoint activation.. J Cell Biol.

[22] Cortez D, Glick G, Elledge SJ (2004). Minichromosome maintenance proteins are direct targets of the ATM and ATR checkpoint kinases.. Proc Natl Acad Sci U S A.

[23] Tsao CC, Geisen C, Abraham RT (2004). Interaction between human MCM7 and Rad17 proteins is required for replication checkpoint signaling.. EMBO J.

[24] Christensen TW, Tye BK (2003). Drosophila MCM10 interacts with members of the prereplication complex and is required for proper chromosome condensation.. Mol Biol Cell.

[25] Woodward AM, Gohler T, Luciani MG, Oehlmann M, Ge X (2006). Excess Mcm2-7 license dormant origins of replication that can be used under conditions of replicative stress.. J Cell Biol.

[26] Forsburg SL (2004). Eukaryotic MCM proteins: beyond replication initiation.. Microbiol Mol Biol Rev.

[27] Schwed G, May N, Pechersky Y, Calvi BR (2002). Drosophila minichromosome maintenance 6 is required for chorion gene amplification and genomic replication.. Mol Biol Cell.

[28] Pflumm MF, Botchan MR (2001). Orc mutants arrest in metaphase with abnormally condensed chromosomes.. Development.

[29] Feger G, Vaessin H, Su TT, Wolff E, Jan LY (1995). dpa, a member of the MCM family, is required for mitotic DNA replication but not endoreplication in Drosophila.. EMBO J.

[30] Labib K, Kearsey SE, Diffley JF (2001). MCM2-7 proteins are essential components of prereplicative complexes that accumulate cooperatively in the nucleus during G1-phase and are required to establish, but not maintain, the S-phase checkpoint.. Mol Biol Cell.

[31] Matsubayashi H, Yamamoto MT (2003). REC, a new member of the MCM-related protein family, is required for meiotic recombination in Drosophila.. Genes Genet Syst.

[32] Takahashi TS, Wigley DB, Walter JC (2005). Pumps, paradoxes and ploughshares: mechanism of the MCM2-7 DNA helicase.. Trends Biochem Sci.

[33] Pacek M, Walter JC (2004). A requirement for MCM7 and Cdc45 in chromosome unwinding during eukaryotic DNA replication.. EMBO J.

[34] Loupart ML, Krause SA, Heck MS (2000). Aberrant replication timing induces defective chromosome condensation in Drosophila ORC2 mutants.. Curr Biol.

[35] Crevel G, Mathe E, Cotterill S (2005). The Drosophila Cdc6/18 protein has functions in both early and late S phase in S2 cells.. J Cell Sci.

